# miR-146a-5p-mediated suppression on trophoblast cell progression and epithelial-mesenchymal transition in preeclampsia

**DOI:** 10.1186/s40659-021-00351-5

**Published:** 2021-09-13

**Authors:** Pingping Peng, Huamei Song, Chenghong Xie, Wenfei Zheng, Huigai Ma, Dandan Xin, Jingqiong Zhan, Xiaoqing Yuan, Aihua Chen, Jing Tao, Jufang Qin

**Affiliations:** 1 Department of Gynecology and Obstetrics, the First People’s Hospital of Yichang, Yichang, 443000 Hubei People’s Republic of China; 2 Department of Gynecology and Obstetrics, the People’s Hospital of China Three Gorges University, Yichang, 443000 Hubei People’s Republic of China; 3 Department of Gynecology and Obstetrics, the First People’s Hospital of Yichang, the People’s Hospital of China Three Gorges University, No. 4, Hudi Street, Xiling District, Yichang, 443000 Hubei People’s Republic of China

**Keywords:** Trophoblast cells, miR-146a-5p, Wnt2, Proliferation, Migration, Epithelial-mesenchymal transition

## Abstract

**Objective:**

This study aims to identify the effect of miR-146a-5p on trophoblast cell invasion as well as the mechanism in preeclampsia (PE).

**Methods:**

Expression levels of miR-146a-5p and Wnt2 in preeclamptic and normal placentae were quantified. Trophoblast cells (HTR-8) were separately transfected with miR-146a-5p mimic, miR-146a-5p inhibitor, pcDNA3.1-Wnt2 or sh-Wnt2, and then the expression levels of miR-146a-5p, Wnt2, and epithelial-mesenchymal transition (EMT)-related proteins (Vimentin, N-cadherin and E-cadherin) were measured. Moreover, the proliferative, migratory and invasive capacities of trophoblast cells were detected, respectively. Dual luciferase reporter assay determined the binding of miR-146a-5p and Wnt2.

**Results:**

Compared with normal placental tissues, the placentae from PE patients showed higher miR-146a-5p expression and lower Wnt2 expression. Transfection of miR-146a-5p inhibitor or pcDNA3.1-Wnt2 exerted pro-migratory and pro-invasive effects on HTR-8 cells and encouraged EMT in HTR-8 cells; transfection with miR-146a-5p mimic or sh-Wnt2 weakened the proliferative, migratory and invasive capacities as well as reduced EMT process of HTR-8 cells. Moreover, Wnt2 overexpression could partially counteract the suppressive effects of miR-146a-5p overexpression on the progression and EMT of HTR-8 cells.

**Conclusion:**

miR-146a-5p mediates trophoblast cell proliferation and invasion through regulating Wnt2 expression.

## Introduction

Preeclampsia (PE) is a syndrome of pregnancy characterized by hypertension and proteinuria [1], which is a dominant cause of maternal and perinatal deaths with the incidence of 5–7% [2]. Although the explicit pathogenesis of PE has not been fully understood, impaired spiral artery remodeling, oxygen dysregulation, and increased maternal vascular damage are admittedly considered causative factors of PE [3]. During the first trimester of pregnancy, invasive extravillous trophoblasts (EVTs) are responsible for the nutrient supply demanded by the fetus through remodeling the uterine spiral arteries [4]. Highly invasive EVTs, derived from trophoblast cells by epithelial-mesenchymal transition (EMT), are able to establish maternal–fetal linkage [5]. In this regard, insufficient acquisition of invasive and migratory capacities by trophoblast cells plays a vital role in the onset of PE. Epithelial-mesenchymal transition (EMT) is a physiological process that gives epithelial cells invasive and migratory potential to become cells with mesenchymal cell morphology and properties [6, 7]. Moreover, dysregulated EMT of trophoblast cells in PE induces defective migration and invasion [5]. These findings emphasize the demand for disclosing the effective regulators of trophoblast cell progression, especially of trophoblast EMT process.

MicroRNAs (miRNAs), a type of non-coding RNAs with  ~  22 nt in length, are endowed with posttranscriptionally regulatory potential through paring with sequence in the 3′untranslated region (UTR) of target genes [8]. Many researches have figured out the important roles of miRNAs in trophoblast cell progression. For instance, miR-134 impairs trophoblast cell infiltration in PE through downregulating Integrin Beta 1 (ITGB1) [9]. miR-616-3p upregulation stimulates trophoblast cell growth and migration in PE through inhibiting tissue factor pathway inhibitor 2 (TFPI2) [10]. As a mature miRNA generated by miR-146a, miR-146a-5p shows suppressive effects on the proliferative, invasive and migratory capacities of breast cancer cells [11] and on the EMT process of oesophageal squamous cells [12]. Upregulated miR-146a-5p expression was found in patients with a history of gestational hypertension [13]. In PE patients, downregulation of miR-146a-5p was reported by Hromadnikova et al. [14]. Ding and colleagues instead demonstrated overexpressed miR-146a-5p in placentae from PE patients [15]. The expression pattern of miR-146a-5p in PE is controversial and its biological functions remain to be investigated. Wnt2, one of the Wnt ligands, is a secreted glycoprotein downregulated in PE placentae [16]. Wnt2 is elucidated to promote the proliferation and migration of first trimester trophoblast cells via activating Wnt/β-catenin pathway [17]. In this study, the online software StarBase predicted the binding sites of miR-146a-5p in the 3′UTR of Wnt2, which implied that the interplay of miR-146a-5p and Wnt2 may play a role in regulating trophoblast cell progression.

In the present study, the expression levels of miR-146a-5p and Wnt2 in the placentae from patients with PE were confirmed. The biological functions of miR-146a-5p/Wnt2 axis were also determined in in vitro experiments. We demonstrated the involvement of miR-146a-5p/Wnt2 axis in the trophoblast cell progression and EMT event, which could provide a latent approach for improving the invasive and migratory capacities of trophoblast cells and mitigating PE development.

## Materials and methods

### Placental tissue collection

Collection of placental tissues from normal pregnant female (n  =  30; Normal group) and patients with PE (n  =  30; PE group) was conducted since January 2018–May 2020 at the First People’s Hospital of Yichang. Ethics Committee approval from the First People’s Hospital of Yichang was obtained after informed written consent was provided by each patient. This study conformed to the principles issued in the Declaration of Helsinki. Clinical data of participants are listed in Table 1. The collected placental tissues were frozen by liquid nitrogen and preserved at − 80 °C before further analyses. The diagnosis criteria for PE were as follows [18]: patients had no history of preexisting or chronic hypertension, but exhibited systolic pressure  >  140 mmHg or diastolic pressure  >  90 mmHg at least 2 occasions, concurrent with proteinuria (>  2 g per 24 h in 2 samples obtained at  >  4 h intervals) after 20 weeks of gestation. The exclusion criteria included chronic hypertension, BMI  >  24 before pregnancy, heart disease, diabetes, hepatopathy, nephropathy, intra uterine fetal death, fetal chromosomal or congenital abnormalities, and pregnancy following fertility treatment.

**Table 1:** Clinical data of normal pregnant women and PE patients

Clinical data	Control (n = 30)	PE (n = 30)	*P* values
Maternal age (year)	30.5 ± 4.8	30.2 ± 5.1	> 0.05
Maternal weight (kg)	68.5 ± 5.9	70.6 ± 6.8	> 0.05
Gestational age (week)	38.7 ± 2.5	36.3 ± 2.5	< 0.05
Systolic blood pressure (mm Hg)	112.5 ± 7.2	170.3 ± 8.9	< 0.001
Diastolic blood pressure (mm Hg)	75.9 ± 2.5	118.7 ± 5.5	< 0.001
Proteinuria (g/day)	Not detected	4.5 ± 1.3	< 0.01

*PE* preeclampsia

### Cell culture

Human trophoblast cell line [HTR-8; American Type Culture Collection (ATCC)] was cultured in RPMI-1640 (Thermo Fisher Scientific, MA, USA) supplemented with 10% fetal bovine serum (FBS; Thermo Fisher Scientific, MA, USA), 100 U/mL penicillin and 100 µg/ml streptomycin. Human embryonic kidney cells (HEK-293 T; ATCC) were grown in DMEM containing 10% FBS, 100 U/mL penicillin and 100 µg/ml streptomycin. HTR-8 and HEK-293 T cells were maintained under humidified conditions (37 °C, 5% CO_2_).

### Transfections

Short hairpin RNA (shRNA) against Wnt2 (sh-Wnt2; 2 µg), pcDNA3.1 plasmid with Wnt2 overexpression (pcDNA3.1-Wnt-2; 2 µg), miR-146a-5p mimic (100 nM), miR-146a-5p inhibitor (100 nM), and their negative controls (sh-NC, pcDNA3.1, mimic NC and inhibitor NC) (RiboBio Co., Ltd., Guangzhou, China) were transfected into HTR-8 cells using Lipofectamine 2000 kit (Thermo Fisher Scientific, MA, USA). These cells were accordingly designated into pcDNA3.1 group, pcDNA3.1-Wnt2 group, sh-NC group, sh-Wnt2 group, mimic NC group, miR-146a-5p mimic group, inhibitor NC group, miR-146a-5p inhibitor group, miR-146a-5p mimic  +  pcDNA3.1 group and miR-146a-5p mimic  +  pcDNA3.1-Wnt2 group. Stably transfected cells were maintained in RPMI-1640 and further cultured at 37 °C and 5% CO_2_.

### MTT assay

Following treatment for 24 h, 48 h, 72 h and 96 h, cells were counted. Cell suspension (100 μL/well, 10^4^–10^5^ cells) was seeded onto a 96 well plate in triplicate, and then the cells were placed at 37 °C and 5% CO_2_. After 20 μL of MTT solution (5 mg/mL, Sigma, MO, USA) were pipetted into the wells, the cells were further cultured for 4 h at 37 °C and 5% CO_2_. The medium was removed and DMSO (150 μL/well) was pipetted onto the cells to dissolve the crystal. The absorbance of each group was measured three times and then averaged. Each group had 3 replicate wells.

### Colony formation assay

For colony formation assay, monolayer cells were digested with 0.25% trypsin, flapped and suspended in 10% FBS. The cell suspension was seeded onto culture plates containing 10 ml of warm (37 °C) culture medium at gradient densities (50, 100 or 200 cells per well), after which the cells was dispersed and cultured at 37 °C under humidified conditions for 2–3 weeks. The incubation was not terminated until colonies could be observed by naked eyes. After the medium was pipetted off, the cells were rinsed twice in PBS and exposed to 5 ml acetic acid/methanol (1:3) for 15 min and Giemsa stain for 10–30 min. Then, the cells were washed to remove the excess stain and air dried. The culture plate was inverted and overlapped by a transparent film with grid. Finally, the colonies (more than 10 cells) were counted by naked eyes or under a low power microscope to calculate the colony formation rate. Each group had 3 replicate wells.

### Transwell invasion assay

Transwells (6.5 mm in diameter; 8 µm pore; Corning Costar, Cambridge, MA) were pre-coated with 0.1 ml matrigel (200 µg/ml; BD Bioscience, Franklin Lakes, NJ, USA) and maintained at 37 ℃ overnight for gelling. Subsequently, stably transfected HTR-8 cells (5  ×  10^4^ cells/well) were resuspended and seeded onto the transwells. RPMI-1640 containing 10% FBS was added into basolateral chamber and cultured (37 ℃, 5% CO_2_) for 24 h. Afterwards, the cells were immersed in 4% paraformaldehyde for 10 min and stained (10 min, room temperature) by 0.5% crystal violet. Invaded cells were photographed under an optical microscope in five random fields. All data were from three independent experiments. Each group was repeated for three times.

### Scratch assay

Scratch assay was conducted as previously described [19]. In brief, HTR-8 cells were seeded in 6-well plates and grew to 90% confluence. Thereafter, three scratches were made in each well vertically using a 100 μL pipette tip, and the cells were washed and cultured in serum free culture medium. A low power phase contrast microscope was used to observe the scratches. After further incubation for 24 h, the scratches were photographed. Migration rate  =  (scratch gap at 0 h − scratch gap at 24 h)/scratch gap at 0 h. Each group had 3 replicate wells.

### RT-qPCR

Following cell transfection, HTR-8 cells were lysed in 1 ml Trizol (Thermo Fisher Scientific, MA, USA) to obtain RNA extracts. After quantification, the RNA extracts were reverse transcribed to synthesize cDNA. Relative mRNA and miRNA expression levels were determined by using a fluorescent quantitative PCR kit (Takara, Dalian, China) on quantitative PCR instrument ABI7500 (Applied Biosystems, Shanghai, China). The reactions were performed under the following conditions: predegradation (95 ℃, 10 min); 40 cycles of degradation (95 ℃, 10 s), annealing (60 ℃, 20 s) and extension (72 ℃, 34 s). All primers used in this work were synthesized by GENEWIZ, Inc. (Beijing, China) (Table 2). For miRNA and mRNA expression analyses, U6 and GAPDH were used as loading controls. Relative expression was measured using 2^−ΔΔCt^ method as previously described [20]. ΔΔCt  =  [Ct_(target gene)_ − Ct_(reference gene)_]_experimental group_ − [Ct_(target gene)_ − Ct_(reference gene)_]_control group_. Each group had 3 replicate wells.

**Table 2:** Names and primer sequences of genes of interest and relative internal references

Name of primer	Sequences(5′-3′)
miR-146a-5p-F	AACCCATGGAATTCAGTTCTCA
miR-146a-5p-R	ATCCAGTGCAGGGTCCGAGG
Wnt2-F	GGGTCCTACTCCGAAGTAG
Wnt2-R	CCTTGGCTACAGGCCCTG
Vimentin-F	TCCGCACATTCGAGCAAAGA
Vimentin-R	TGAGGGCTCCTAGCGGTTTA
N-cadherin-F	AGGGGAGAGGTGCTCTACTG
N-cadherin-R	GGGGTAATCCACACCACCTG
E-cadherin-F	CGTCGAGCTCTTGACCGAAA
E-cadherin-R	TCAAACACCTCCTGTCCTCT
GAPDH-F	ACCACAGTCCATGCCATCAC
GAPDH-R	TCCACCACCCTGTTGCTGTA
U6-F	TCGCTTCGGCAGCACATATAC
U6-R	GCGTGTCATCCTTGCGCAG

*F* forward; *R* reverse

### Western blotting

Placental tissues and HTR-8 cells were washed twice with ice-cold PBS, and total protein extracts were obtained from HTR-8 cells lysed in protein extraction lysate (100 μL/50 mL). Then, the lysed cell was maintained on ice for 30 min and centrifuged (12,000 rpm, 10 min). The supernatant were aliquoted into 0.5 ml centrifuge tubes and preserved at − 20 °C or quantified by BCA kit (Sigma-Aldrich, St. Louis, MI, USA). The protein extracts were degraded in 6  ×  SDS loading buffer at 100 ℃ and separated by SDS electrophoresis. Afterwards, the protein extracts were transferred to membranes in ice-cold transfer buffer (4 ℃) for 1.5 h, ant the membranes were immersed in TBST supplemented with 5% non-fat milk powder for 1 h. The membranes were further incubated in TBST containing primary antibodies against rabbit derived GAPDH (1:10,000, ab181602), Wnt2 (1:500, ab27794), Vimentin (1:2000, ab92547), N-cadherin (1:1000, ab 76,057), and E-cadherin (1:1000, ab40772) (Abcam, MA, USA) overnight at 4 ℃. After TBST washing (3  ×  10 min), the membranes were incubated (2 h, room temperature) with goat anti rabbit IgG or goat anti mouse IgG (Beijing ComWin Biotech Co., Ltd., Beijing, China). Following TBST washing and color development, protein expression of genes of interest was quantified. Each group was repeated for 3 times.

### Dual luciferase reporter assay

The binding sites of miR-146a-5p in the 3′UTR of Wnt2 were predicted by online software StarBase. Accordingly, the wild and mutated sequences of the binding sites were synthesized and cloned into luciferase reporter vectors (Promega) to construct the vectors WT-Wnt-2 and MUT-Wnt-2. Then, the WT-Wnt2 and MUT-Wnt2 were separately cotransfected with miR-146a-5p mimic or miR-146a-5p inhibitor into HEK-293 T cells. These cells were further cultured (48 h, 37 ℃, 5% CO_2_) in DMEM, and luciferase activity in each group was determined using a luciferase reporter assay kit (Promega, WI, USA). Each group had 3 replicate wells.

### Statistical analysis

Data analyses were conducted by statistical software SPSS 18.0 (IBM Corp., Armonk, NY, USA) and GraphPad Prism 6.0 (GraphPad Software Inc.) and presented as mean  ±  standard deviations (SDs). *T *test was used for comparisons between two groups and one-way analysis of variance was performed for multiple group analyses. Results were considered significant when *P*  <  0.05. Each experiment was repeated for three times.

## Results

### High miR-146a-5p expression and low Wnt2 expression in the placentae from PE patients

**Fig. 1:**
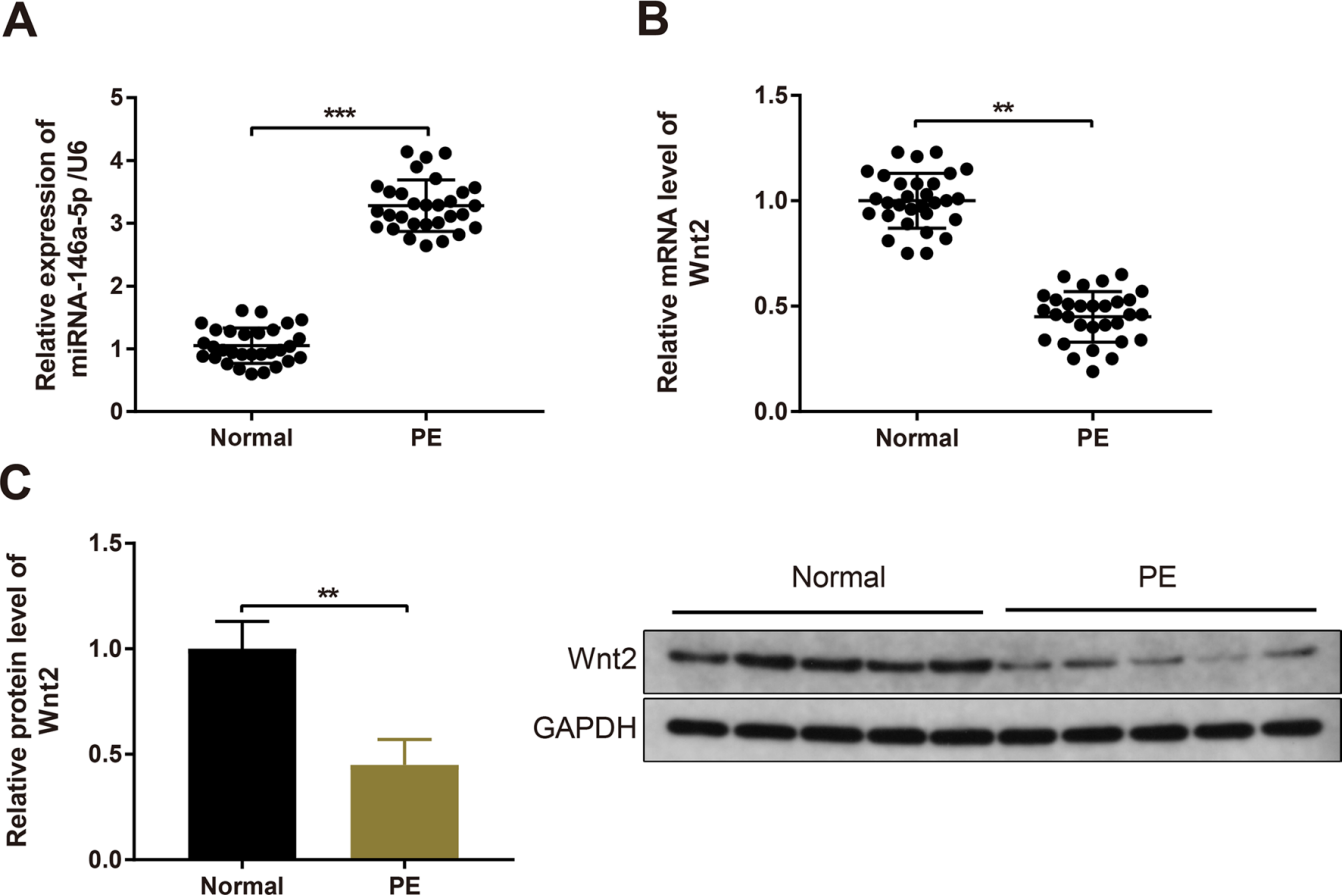
Upregulated miR-146a-5p expression and downregulated Wnt2 expression in the placentae from PE patients. Note: RT-qPCR was used to measure miR-146a-5p expression in the placentae from normal and PE patients (**A**); Wnt2 expression was determined by RT-qPCR (**B**) and Western blotting (**C**). All data are presented as mean  ±  SDs; n  =  30, **P  <  0.01, ***P  <  0.001. *SD* standard deviation; *PE* preeclampsia

Initially, the clinicopathological characteristics of 30 normal pregnant women and 30 PE patients were collected and analyzed (Table 1). Analyses of the clinical data in the PE and normal groups revealed that both the systolic and diastolic blood pressures were elevated in the PE group; proteinuria was detected in the PE group, whereas it was not assessed in the normal group (Table 1). Moreover, the gestational age in the PE group was significantly decreased than that in the normal group (Table 1). Furthermore, the expression levels of miR-146a-5p and Wnt2 in placentae from PE and normal pregnant females were assayed. RT-qPCR revealed that miR-146a-5p expression was increased in placentae from PE patients (Fig. 1A, *P*  <  0.001). As shown by RT-qPCR and Western blotting, the expression of Wnt2 mRNA and protein was decreased in the PE patients compared with normal pregnant women (Fig. 1B, C, *P * <  0.01).

### miR-146a-5p affects trophoblast cell propagation, invasion and EMT

**Fig. 2:**
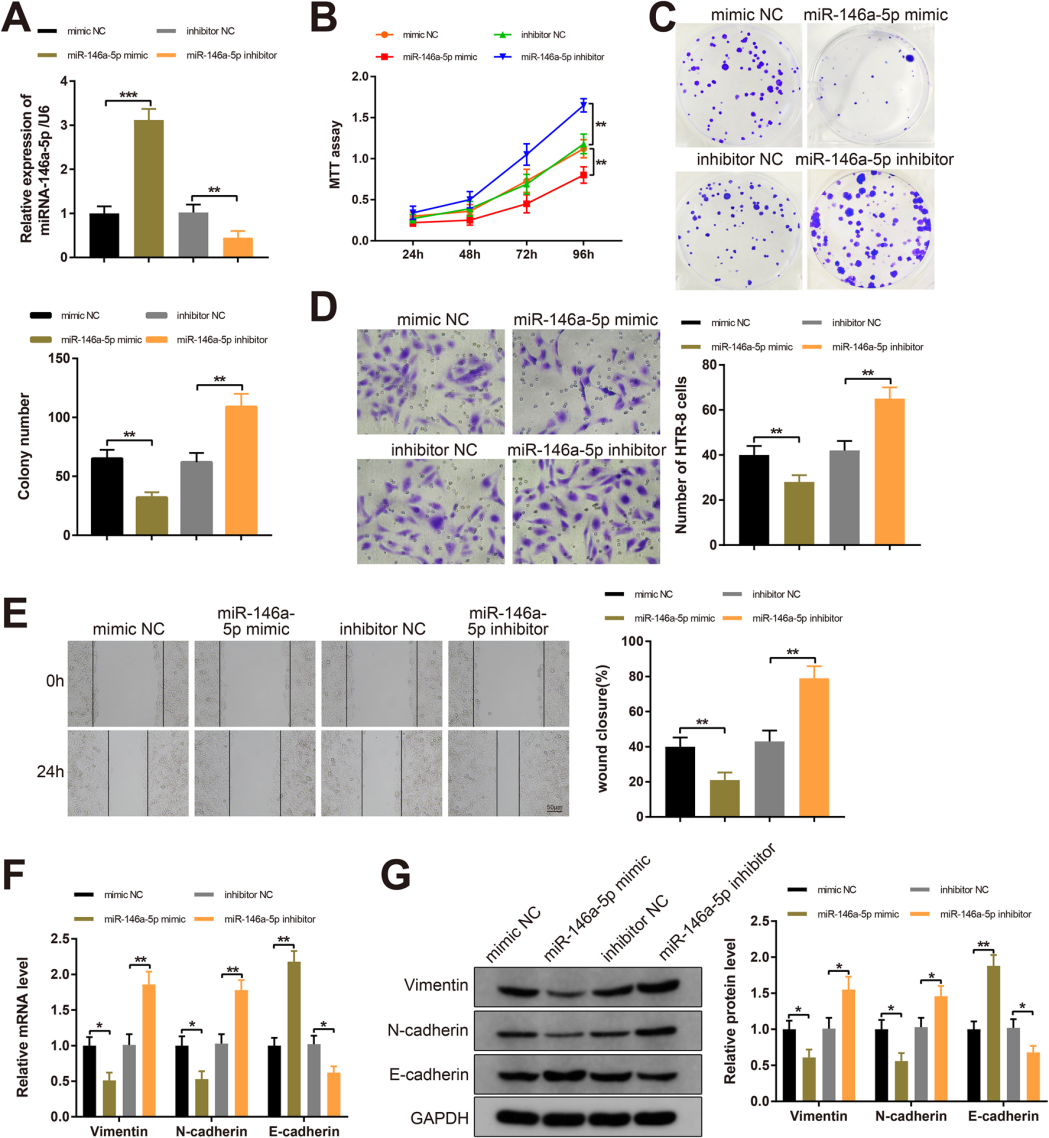
miR-146a-5p inhibits biological properties and EMT of trophoblast cells. Note: After HTR-8 cells were transfected with miR-146a-5p mimic, miR-146a-5p inhibitor, mimic NC or inhibitor NC, RT-qPCR was used to detect miR-146a-5p expression (**A**); MTT assay (**B**) and colony formation assay (**C**) determined the proliferative capacity of HTR-8 cells; invasiveness of HTR-8 cells as measured by Transwell invasion assay (**D**); HTR-8 cell migration was evaluated by Scratch assay (**E**); RT-qPCR and Western blotting examined the expression levels of Vimentin, N-cadherin and E-cadherin (**F**, **G**). All data are presented as mean  ±  SDs; n  =  3, *P  <  0.05, **P  <  0.01, ***P  <  0.001. *SD* standard deviation; *EMT* epithelial-mesenchymal transition

To identify the biological functions of miR-146a-5p in trophoblast cells, miR-146a-5p was overexpressed or silenced in HTR-8 cells. RT-qPCR analysis showed higher miR-146a-5p level in the miR-146a-5p mimic group (*P*  <  0.001, vs mimic NC group) and lower miR-146a-5p expression in the miR-146a-5p inhibitor group (*P*  <  0.01, vs inhibitor NC group), whereas the difference in miR-146a-5p expression between the mimic NC group and the inhibitor NC group was indistinctive (Fig. 2A). The results indicated the satisfactory transfection of miR-146a-5p mimic and miR-146a-5p inhibitor.

To determine the impact of miR-146a-5p on the proliferative, migratory and invasive capacities of HTR-8 cells, MTT, colony formation, Transwell invasion and Scratch assays were performed. Compared with the mimic NC group, the proliferative capacity of HTR-8 cells was inhibited and the number of colonies was reduced in the miR-146a-5p mimic group (Fig. 2B, C, *P*  <  0.01). Moreover, the invasive and migratory capacities were reduced in the miR-146a-5p mimic group compared with the mimic NC group (Fig. 2D, E, *P * <  0.01). Furthermore, the miR-146a-5p inhibitor group showed increased proliferative, invasive and migratory capacities compared to the inhibitor NC group (Fig. 2B–E, *P*  <  0.01). These observations revealed that miR-146a-5p suppressed the proliferative, migratory and invasive capacities of trophoblast cells.

EMT process reportedly involves in the initiation and development of various human diseases, including PE [21], mainly manifesting as decreased expression of epithelial markers (i.e., E-cadherin) and increased expression of mesenchymal markers (i.e., N-cadherin and Vimentin) [22]. During the process, E-cadherin is converted to N-cadherin, thereby enhancing cell invasion and migration [23]. As detected by RT-qPCR and Western blotting, Vimentin and N-cadherin levels were reduced and E-cadherin expression was elevated in the miR-146a-5p mimic group compared with mimic NC group (Fig. 2F, G, *P * <  0.05). Conversely, the levels of Vimentin and N-cadherin were increased and E-cadherin was decreased in the miR-146a-5p inhibitor group (Fig. 2F, G, *P * <  0.05, vs the inhibitor NC group). Taken together, miR-146a-5p suppressed the proliferation, migratory and invasive properties as well as EMT of trophoblast cells.

### Wnt2 augments the proliferation, invasion and EMT process of trophoblast cells

**Fig. 3:**
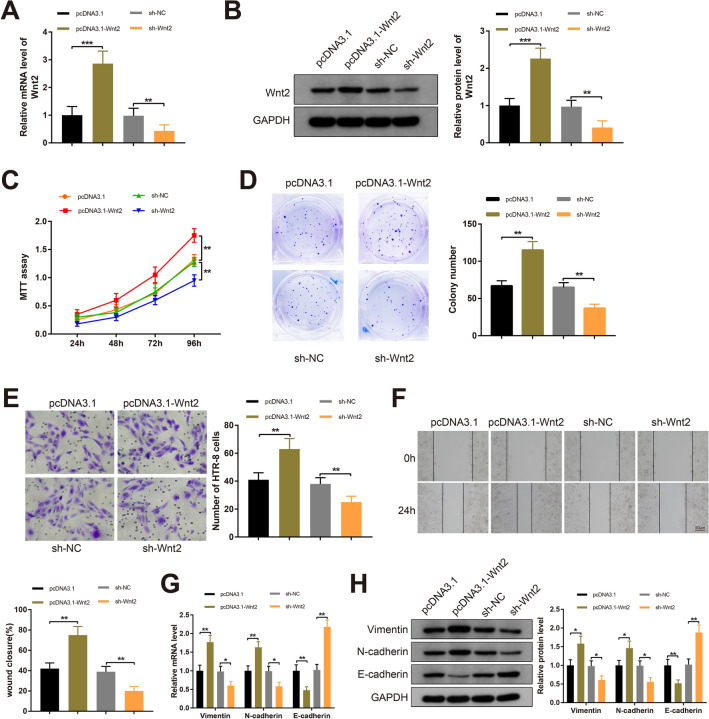
Wnt2 promotes the proliferation, migration, invasion and EMT of trophoblast cells. Note: pcDNA3.1-Wnt2, sh-Wnt2, pcDNA3.1 or sh-NC was transfected into HTR-8 cells, and then Wnt2 expression was determined by RT-qPCR (**A**) and Western blotting (**B**); the proliferative capacity of HTR-8 cells as measured by MTT assay (**C**) and colony formation assay (**D**); Transwell invasion assay (**E**) and Scratch assay (**F**) assessed the migratory capacity and invasiveness of HTR-8 cells separately; RT-qPCR (**G**) and Western blot (**H**) analyses for EMT-related proteins (Vimentin, N-cadherin and E-cadherin). All data are presented as mean  ±  SDs; n  =  3, *P  <  0.05, **P  <  0.01, ***P  <  0.001. *SD* standard deviation; *EMT* epithelial-mesenchymal transition

pcDNA3.1-Wnt2 and sh-Wnt2 were introduced into HTR-8 cells to investigate the effect of Wnt2 on trophoblast cells. Transfection of pcDNA3.1-Wnt2 increased Wnt2 expression in HTR-8 cells compared to those transfected with pcDNA3.1, and HTR-8 cells transfected with sh-Wnt2 showed decreased Wnt2 expression compared with the sh-NC group (Fig. 3A, B, *P*  <  0.01), indicating satisfactory transfection of pcDNA3.1-Wnt2 and sh-Wnt2 in HTR-8 cells.

As exhibited in Fig. 3C, D, the proliferation of HTR-8 cells was enhanced in the pcDNA3.1-Wnt2 group (vs the pcDNA3.1 group), while Wnt2 silencing induced a decrease in HTR-8 cell proliferation (vs the sh-NC group) (*P * <  0.01). Transwell invasion and Scratch assays revealed increased migratory and invasive capacities in the pcDNA3.1-Wnt2 group compared to the pcDNA3.1 group, whereas the migration and invasiveness of HTR-8 cells were reduced after transfection with sh-Wnt2 (*P*  <  0.01); significant difference in the migration and invasiveness of HTR-8 cells was found neither in the pcDNA3.1 nor in the sh-NC groups (Fig. 3E, F). Moreover, RT-qPCR and Western blot analyses exhibited great increases in Vimentin and N-cadherin levels and a reduction in E-cadherin expression in the pcDNA3.1-Wnt2 group compared with the pcDNA3.1 group, and conversely, transfection with sh-Wnt2 decreased Vimentin and N-cadherin levels and increased E-cadherin expression in HTR-8 cells when compared with the sh-NC group (Fig. 3G, H, *P*  <  0.01). These results were indicative of the enhancement of Wnt2 on the proliferative, migratory and invasive capacities and EMT process of trophoblast cells.

### Wnt2 is a target of miR-146a-5p

**Fig. 4:**
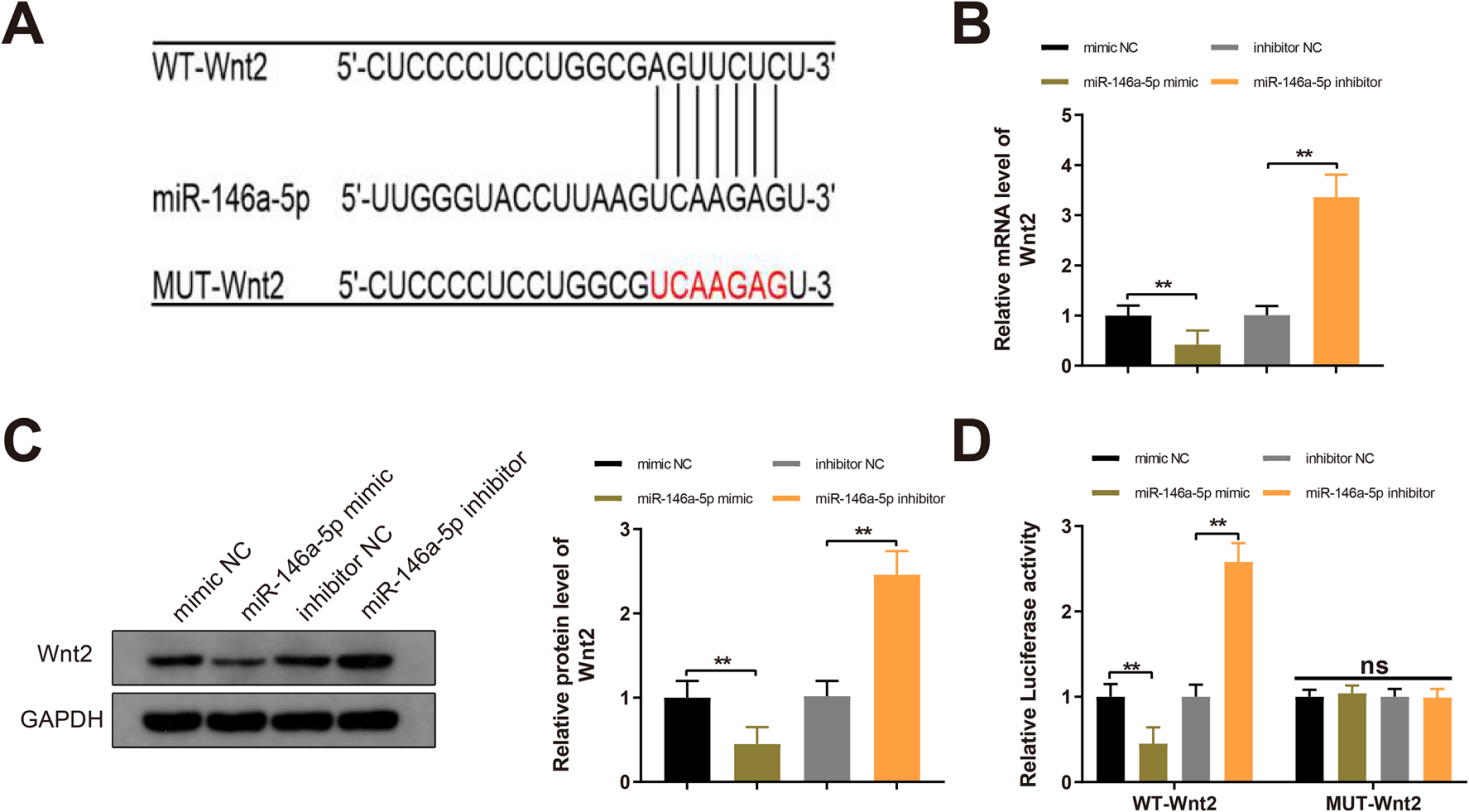
miR-146a-5p targets Wnt2. Note: The binding sites of miR-146a-5p in the 3′UTR of Wnt2 were predicted by StarBase and the corresponding mutated target sites were designed (**A**). Following transfection of miR-146a-5p mimic, miR-146a-5p inhibitor, mimic NC or inhibitor NC into HTR-8 cells, the mRNA and protein levels of Wnt2 in HTR-8 cells were quantified by RT-qPCR and Western blotting (**B**, **C**). HEK-293 T cells were cotransfected with miR-146a-5p mimic or miR-146a-5p inhibitor and MUT-Wnt2 or WT-Wnt2, and the luciferase activity was determined by dual luciferase reporter assay (**D**). All data are presented as mean  ±  SDs; n  =  3, **P  <  0.01. *SD* standard deviation; *3′UTR* 3′untranslated region

Online software StarBase probed the binding sites of miR-146a-5p in the 3’UTR of Wnt2 (Fig. 4A). Hence, we hypothesized that miR-146a-5p implicated in the biological properties of trophoblast cells through regulating Wnt2. To test this hypothesis, miR-146a-5p was overexpressed or inhibited in HTR-8 cells. In the miR-146a-5p mimic group, the expression of Wnt2 mRNA and protein was reduced compared to the mimic NC group; compared to the inhibitor NC group, Wnt2 expression was enhanced in the miR-146a-5p inhibitor group (Fig. 4B, C, *P*  <  0.01). To investigate whether miR-146a-5p could bind to Wnt2, WT-Wnt2 and MUT-Wnt2 (containing 7 mutated binding sites of miR-146a-5p in the 3’UTR of Wnt2) were constructed. The dual luciferase reporter assay suggested that cotransfection of WT-Wnt2 and miR-146a-5p mimic decreased the luciferase activities of HEK-293 T cells (*P*  <  0.01, vs the mimic NC group); conversely, HEK-293 T cells cotransfected with WT-Wnt2 and miR-146a-5p inhibitor showed increased luciferase activity (*P*  <  0.01, vs the inhibitor NC group); the luciferase activities of cells cotransfected with MUT-Wnt2 and miR-146a-5p mimic/inhibitor displayed no significant difference when compared with the mimic NC or inhibitor NC group (Fig. 4D). Together, miR-146a-5p targeted and downregulated Wnt2.

### miR-146a-5p regulates the propagation, invasion and EMT of trophoblast cells through Wnt2

**Fig. 5:**
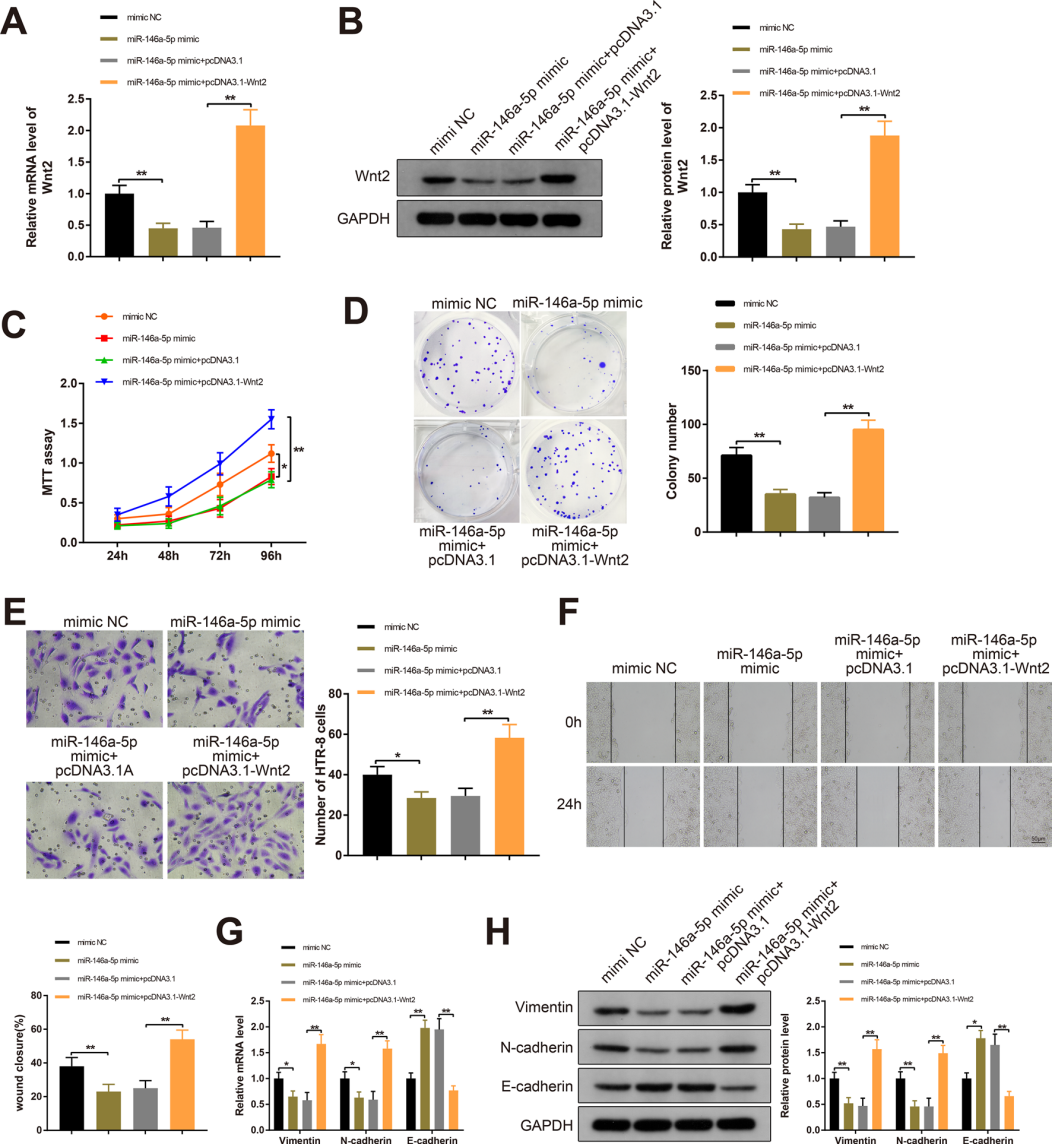
miR-146a-5p regulates Wnt2 to affect trophoblast cell progression and EMT process. Note: After HTR-8 cells were transfected or cotransfected with miR-146a-5p mimic or pcDNA3.1-Wnt2, the mRNA and protein levels of Wnt2 were measured by RT-qPCR (**A**) and Western blotting (**B**); MTT assay and colony formation assay detected the proliferation of HTR-8 cells (**C**, **D**); invasiveness of HTR-8 cells as measured by Transwell invasion assay (**E**); HTR-8 cell migration was assessed by Scratch assay (**F**); RT-qPCR and Western blot analyses for EMT-related proteins (**G**, **H**). All data are presented as mean  ±  SDs; n  =  3, *P  <  0.05, **P  <  0.01. *SD* standard deviation; *EMT* epithelial-mesenchymal transition

To explore the implication of miR-146a-5p/Wnt2 axis in trophoblast cells, miR-146a-5p and/or Wnt2 was overexpressed in HTR-8 cells through transfection or cotransfection with mimic NC, miR-146a-5p or pcDNA3.1-Wnt2. As measured by RT-qPCR and Western blotting, Wnt2 expression in the miR-146a-5p mimic  +  pcDNA3.1-Wnt2 group was upregulated compared to the miR-146a-5p mimic  +  pcDNA3.1 group (*P*  <  0.01); compared with the mimic NC group, Wnt2 expression was reduced in the miR-146a-5p mimic group (*P*  <  0.01); while significant difference in Wnt2 expression was detected neither in the miR-146a-5p mimic group nor in the miR-146a-5p mimic  +  pcDNA3.1 group (Fig. 5A, B).

The proliferation of HTR-8 cells in the miR-146a-5p mimic  +  pcDNA3.1-Wnt2 group was enhanced compared to the miR-146a-5p mimic  +  pcDNA3.1 group (*P*  <  0.01); in the miR-146a-5p mimic group, the proliferative capacity and the number of colonies were decreased than those in the mimic NC group (*P*  <  0.05) (Fig. 5C, D). Also, the migratory and invasive capacities of HTR-8 cells cotransfected with miR-146a-5p mimic and pcDNA3.1-Wnt2 were promoted compared to the miR-146a-5p mimic  +  pcDNA3.1 group (*P * <  0.01), and cells in the miR-146a-5p mimic group showed inhibited migratory capacity and invasiveness compared to the mimic NC group (*P*  <  0.05) (Fig. 5E, F). The differences in the malignant behaviors of HTR-8 cells were indistinctive between the miR-146a-5p mimic group and miR-146a-5p  +  pcDNA3.1 group.

As revealed by RT-qPCR and Western blot analyses, Vimentin and N-cadherin levels were increased and E-cadherin expression was decreased in the miR-146a-5p mimic  +  pcDNA3.1-Wnt2 group (*P*  <  0.01), and the levels of these EMT-related proteins were changed insignificantly in the miR-146a-5p mimic group, compared with the miR-146a-5p mimic  +  pcDNA3.1 group (Fig. 5G, H). These results indicated that Wnt2 overexpression could partially counteract the suppressive effects of miR-146a-5p mimic on HTR-8 cell propagation, invasion and migration as well as EMT process. Collectively, miR-146a-5p regulated trophoblast cell progression and EMT process through Wnt2.

## Discussion

Recently, considerable attention has been paid on the regulation of miRNAs on trophoblast cell invasion and migration in PE [24, 25]. However, the golden standard for PE treatment is still unavailable. To this end, we adopt this study to validate whether the regulatory mechanism of trophoblast cell migration, invasion and EMT in PE is involved with miR-146a-5p/Wnt2 axis.

During embryo implantation, cytotrophoblasts invade the maternal decidua reaching the uterine arteries where they differentiate to an endothelial-like phenotype replacing the maternal vascular endothelial and smooth muscle cells [26]. Failure of trophoblast cells to adequately invade and remodel spiral arteries might induce PE [27]. EMT is a biological process featured by the breakdown of cell–cell adhesion, loss of epithelial phenotypes and cell depolarization [28], and is responsible for normal morphogenetic processes including embryonic development, remodeling and would repair [29]. E-cadherin is a marker of EMT and an integral composition of cell–cell adhesion [29]. Invasion of trophoblast cells to the interstitial tissue of the decidua manifests as a loss of E-cadherin [30]. Intriguingly, miR-146a-5p is well documented as a regulator of cell invasive and migratory capacities. Reportedly, it inhibits cancer cell migration and invasion, impairs the expression of mesenchymal markers (Vimentin, N-cadherin and fibronectin) and promotes the expression of epithelial marker E-cadherin [31]. miR-146a-5p presents anti-migratory effect on prostate cancer cells partially through targeting Rac1 [32]. More importantly, endogenous miR-146a-5p expression was detected in HTR-8 cells [15, 33]. Overexpression of miR-146a decreased the number of invaded trophoblast cells [34]. miR-146a-5p could be transferred by extracellular vesicles secreted by M1 macrophages into trophoblast cells to suppress cell migration and invasion by targeting TRAF6 [15]. Conversely, decreased miR-146a-5p expression was reported to be associated with PE requiring termination of gestation before 34 weeks [14]. These findings indicate that the genetic polymorphism of miR-146a-5p might be related to PE severity. In this paper, miR-146a-5p was upregulated in the placentae from PE patients. Moreover, we demonstrated that miR-146a-5p overexpression induced reductions in the proliferative, migratory and invasive capacities of trophoblast cells. In addition, the trophoblast cells presented augmented E-cadherin expression and weakened Vimentin and N-cadherin expression after miR-146a-5p overexpression. Collectively, we confirmed that miR-146a-5p exhibited suppressive functions on trophoblast cell invasion, migration and EMT. Mechanistically, sirtuin 2 (SIRT2) deacetylated p65 to suppress miR-146a expression, thereby increasing the proliferation, invasion and migration of trophoblast cells [35]. TNF-related apoptosis-inducing ligand (TRAIL) induced trophoblast cell invasion through downregulating miR-146a and upregulating CXCR4, EGFR and matrix metalloproteinase 2 (MMP2) [34]. Of note, inflammation, hypoxia and oxidative stress has been revealed to affect trophoblast invasion [36–38]. Dysregulated miR-146a expression was noticed in patients with recurrent pregnancy loss, concurrent with increased inflammatory and oxidative stress responses [39], and it could attenuate oxygen–glucose deprivation/reoxygenation-induced cardiomyocyte apoptosis [40]. Therefore, it could be an interesting topic to evaluate the impact of miR-146a-5p on trophoblast cell invasion from the perspectives of inflammatory, hypoxia and oxidative stress.

Wnt2, a secreted glycoprotein that functions through autocrine or paracrine modes, is found to express in villous syncytiotrophoblast and EVT, especially in the former [41]. Ye et al. [42] uncovered that Bisphenol A exposure disrupted trophoblast cell invasion and intervened the placental vessel remodeling, consequently leading to PE-like characteristics in pregnant mice, via mediating Wnt2 expression. Herein, our data confirmed a decrease in Wnt2 expression in placental tissues from PE patients. Upregulated Wnt2 enhanced the proliferative, migratory and invasive capacities and promoted the EMT process in trophoblast cells, and silencing of Wnt2 presented converse functions on the trophoblast cells. Consistently, Zhou et al. [43] demonstrated that Wnt2 expression was positively related to metastasis and EMT in cervical cancer. Moreover, this study further identified the binding relationship between miR-146a-5p and Wnt2 by employing bioinformatics analysis and dual luciferase reporter assay. Specifically, miR-146a-5p bound to the 3’UTR of Wnt2 and downregulated Wnt2 expression. Further gain- or loss-of-function assays exhibited that Wnt2 upregulation rescued the invasive and migratory properties of trophoblast cells in the presence of miR-146a-5p overexpression. It could be concluded that Wnt2 overexpression neutralized, in part, the suppressive effects of miR-146a-5p on trophoblast cell proliferation and invasion. Long noncoding RNA AV310809 was demonstrated to promote the EMT process of human peritoneal mesothelial cells through activating the Wnt2/β-catenin pathway by targeting β-catenin [44]. In the transduction of canonical Wnt/β-catenin pathway, Wnt protein activation results in translocation of β-catenin, a subunit of the cell surface cadherin protein complex, into the nucleus, and in turn, it acts as a co-factor of transcription factors [45]. Wnt/β-catenin pathway reportedly regulates morphogenesis, gene transcription, differentiation and proliferation in cells [46]. Slug, Vimentin, MMPs and other downstream genes of Wnt/β-catenin pathway are considered key players in EMT process [47]. In the process of early metastasis, cancer cells undergo EMT event regulated by Wnt/β-catenin pathway [48]. Zhang and colleagues pointed out that forkhead box protein H1 (FOXH1) could encourage EMT through activating the Wnt/β-catenin pathway, thus promoting lung cancer progression [49]. Moreover, resveratrol might induce EMT in trophoblast cells through the Wnt/β-catenin pathway to increase trophoblast cell invasion [28]. It is reasonable to speculate that Wnt2 might induce the translocation of β-catenin into the nucleus of trophoblast cells, while it requires more extensive experiments to identify this hypothesis.

## Conclusion

Our data manifested that miR-146a-5p was upregulated in the preeclamptic placentae. Through in vitro function assays, miR-146a-5p was proven to impair trophoblast cell proliferation, invasiveness and migratory capacity through inhibiting Wnt2. Further investigations regarding the role of miR-146a-5p in the initiation of PE would be anticipated. This study with respect to miR-146a-5p-mediated trophoblast cell invasive and migratory capacities might provide novel insight into the diagnosis and treatment of PE.

## Data Availability

The datasets used or analyzed during the current study are available from the corresponding author on reasonable request.
